# Clinical and Paraclinical Characteristics Relevant to NeuroRehabilitation and Their Outcomes in Postoperative Glioblastoma Patients: A PRISMA Systematic Literature Review

**DOI:** 10.3390/life16071092

**Published:** 2026-06-29

**Authors:** Andreea-Valentina Suciu, Gelu Onose, Constantin Munteanu, Aniela Nodiți-Cuc, Andreea-Iulia Vlădulescu-Trandafir, Cristina Popescu, Ligia-Gabriela Tătăranu

**Affiliations:** 1 Faculty of Medicine, University of Medicine and Pharmacy “Carol Davila”, 020022 Bucharest, Romania; andreea-valentina.spiroiu@drd.umfcd.ro (A.-V.S.); gelu.onose@umfcd.ro (G.O.); cristina_popescu@umfcd.ro (C.P.); ligia.tataranu@umfcd.ro (L.-G.T.); 2 The Neuromuscular Rehabilitation Clinic Division, Teaching Emergency Hospital “Bagdasar-Arseni”, 041915 Bucharest, Romania; constantin.munteanu.biolog@umfiasi.ro; 3 Faculty of Medical Bioengineering, University of Medicine and Pharmacy “Grigore T. Popa” Iasi, 700454 Iași, Romania; 4 Institute of Oncology “Al. Trestioreanu” Bucharest, 022328 Bucharest, Romania; 5 Neurosurgery Clinic Division, Teaching Emergency Hospital “Bagdasar-Arseni”, 041915 Bucharest, Romania

**Keywords:** glioblastoma (GBM), neurorehabilitation, neuro-oncology, functional outcomes, targeted therapies

## Abstract

Background: Glioblastoma (used to be called glioblastoma multiforme—GBM) is the most common and aggressive brain tumor, having the lowest overall survival rate. Initial focal neurological deficits are primarily attributable to surrounding edema; however, as tumor invasion progresses, these deficits become more pronounced and permanent. The standard treatment for newly diagnosed glioblastoma is represented by cytoreductive neurosurgery followed by the Stupp Protocol. Postoperative recovery of the patient with glioblastoma is a long-term process that should include, for overall more acceptable outcomes, neurorehabilitation. This review aims to bring together evidence from neuro-oncology, neurosurgery, and neurorehabilitation in order to better understand the factors associated with recovery, functional status, and quality of life (QoL) after glioblastoma surgery. Our work also aimed to update the related knowledge base and to attempt to optimize the related protocols in patients with operated cerebral glioblastoma. Methods: For these purposes, we conducted a systematic literature review to assess the current state of research referring to the above-mentioned topic. We have used the Preferred Reporting Items for Systematic Reviews and Meta-Analyses (PRISMA—widely recognized internationally) methodology. We used, in this respect, specific keyword combinations/“syntaxes” for searching literature in the domain, in four international databases. Results: Following PRISMA screening, 14 studies met the predefined eligibility criteria. Additional manual reference screening and complementary searches identified further relevant publications, resulting in a total of 22 included articles. Together, the reviewed work addressed a diverse range of topics relevant to postoperative glioblastoma management, including the potential role of multidisciplinary rehabilitation, cognitive interventions, neuromodulation approaches, and functional assessment strategies in improving postoperative outcomes and QoL in glioblastoma patients, while emphasizing that this interdisciplinary domain warrants more extended approaches. Discussion and Conclusions: Despite the relatively limited and largely exploratory available information, neurorehabilitation may contribute to improved functional outcomes and QoL in patients with glioblastoma.

## 1. Introduction

According to the World Health Organization (WHO) classification, there are over one hundred known types of brain and central nervous system tumors [1]. Among these, neoplasms have an aggressive biological behavior and unfavorable clinical outcome. Most of these cancers emerge sporadically, but increasing evidence suggests the involvement of hereditary susceptibility in tumor initiation, particularly in high-grade gliomas, including glioblastoma [2,3].

Glioblastoma is the most common cerebral malignancy, accounting for 10–15% of all brain tumors, and over 50% of all glial tumors. It has the highest grade of malignancy (WHO grade IV) with a median overall survival of 14.6 months, and a 5-year survival rate of 9.8% (which decreases after the age of 65). It can occur at any age, with the highest frequency between 75 and 84 years old [4,5,6,7].

In current practice, the terms “primary” (or “de novo”) and “secondary” glioblastoma (which arise from the malignant transformation of a WHO grade II or III astrocytoma) are used. Primary glioblastomas typically occur in elderly patients and are diagnosed after a short symptom history of approximately 3 months, whereas secondary ones are observed in younger patients and tend to have a more prolonged clinical course [4,8].

Recently, emphasis has been placed on the molecular profile, which supports the production of targeted pathogenic therapies. Regarding isocitrate dehydrogenase (IDH) status, approximately 90% of glioblastomas are IDH wild-type, corresponding to primary glioblastoma, and are associated with a poor prognosis. Conversely, about 10% of glioblastomas are IDH-mutant, corresponding to secondary ones, and have a better prognosis. There are also glioblastomas for which the IDH status cannot be determined. Equally important is the O6-Methylguanine-DNA Methyltransferase (MGMT) status and the presence of the related 1p/19q deletion, which influence the tumor’s response to chemotherapy [4,8,9].

The clinical presentation of patients with glioblastoma is highly diverse, ranging from headache, nausea, and vomiting to motor deficits, epileptic seizures, neuropsychiatric disorders of organic origin, and behavioral, memory, language, and visual impairments [10]. Focal neurological deficits depend on tumor location, resulting in characteristic syndromes that are topographically relevant. These deficits initially arise due to surrounding edema, which is reversible after treatment. However, as tumor invasion progresses, these deficits become more pronounced and even permanent [4].

The standard treatment for newly diagnosed glioblastoma consists of cytoreductive neurosurgery followed by the protocol developed by Dr. Roger Stupp in collaboration with the European Organization for Research and Treatment of Cancer (EORTC) in 2005 [4,11]. The Stupp Protocol consists of radiotherapy (50–60 Gy) and concurrent chemotherapy, followed by an adjuvant chemotherapy—6 months—with Temozolomide (TMZ) [4]. Research into novel treatment modalities focuses on the molecular profiling of glioblastomas, gene therapy, and immunotherapy, all focused on improving patient prognosis [12].

Postoperative recovery in patients with glioblastoma should focus on improving continuity of care, functional recovery, symptom control, and QoL. Rehabilitation programs may involve pain management (including physical therapy), prevention/early detection of complications, rehabilitative kinesiotherapy (mobility training, exercises aimed at improving strength and endurance and aerobic exercises), physiotherapy, nursing, occupational and possibly speech therapy and/or swallowing retraining, as well as psychological support. Postoperative recovery is a prolonged and continuous process; therefore, the patient requires close surveillance and periodic assessments to monitor health status and facilitate early detection of potential recurrences and complications [13,14,15].

In light of the existing knowledge, this systematic review aimed to identify and summarize clinical and paraclinical factors relevant to neurorehabilitation in postoperative glioblastoma patients and to examine the outcomes reported in the current literature. By bringing together these data, we sought to provide an updated overview of the field and to identify aspects that may be relevant for future rehabilitation strategies and clinical practice. In addition to rehabilitation interventions themselves, this review examined clinical and paraclinical factors that may influence rehabilitation planning, functional outcomes, and recovery trajectories in postoperative glioblastoma patients.

## 2. Materials and Methods

### 2.1. Study Design and Research Question

In order to identify the current state of research and the level of knowledge regarding the chosen topic, a systematic literature review was conducted. As a method of selection and filtering of relevant material, the well-known methodology PRISMA was used, enhanced through the use of a customized algorithm for weighted quantification of results [16,17], described in Section 2.4.

This systematic review addressed a detailed PICO question.

The PICO Framework is described below:

Population (P): Adult patients (aged ≥ 18 years) with a diagnosis of cerebral glioblastoma who underwent surgical resection (craniotomy with tumor excision), regardless of adjuvant oncological treatment.

Intervention (I): Neurorehabilitation programs conducted after surgery, as well as prehabilitation strategies, including but not limited to: physical therapy, occupational therapy, speech therapy, cognitive rehabilitation, neuromodulation techniques (e.g., repetitive Transcranial Magnetic Stimulation (rTMS), rehabilitation nursing, and multidisciplinary functional recovery programs.

Comparison (C): Postoperative routine management, either with or without neurorehabilitation intervention (based on study design).

Outcomes (O): Main outcomes include functional recovery, improvement in motor and language functions, cognitive skills, and health-related QoL.

Secondary outcomes include complication rate, length of hospital stay, functional independence recovery, patient survival when provided in connection with rehabilitation programs, and neuroplasticity imaging or electrophysiological markers.

Hence, the PICO question is as follows: In adults who have undergone surgery for cerebral glioblastoma (P), does postoperative neurorehabilitation (I) compared with conventional care or no rehabilitation (C) lead to better functional, cognitive, and/or QoL outcomes (O)?

### 2.2. Search Strategy

The literature search was conducted using keyword combinations/“syntaxes” (Table 1), in the following international databases: Elsevier [18] (last accessed on 2 April 2026), PubMed [19] (last accessed on 2 April 2026), PubMed Central (PMC), [19] (last accessed on 2 April 2026), and Physiotherapy Evidence Database (PEDro) [20], (last accessed on 2 April 2026).

### 2.3. Eligibility Criteria

According to the PRISMA methodology, specific eligibility criteria were applied. We included studies that (1) reported original clinical research (prospective or retrospective observational studies, interventional trials) or systematic and/or narrative reviews, or meta-analyses; (2) involved adult patients (≥18 years old) with histologically confirmed cerebral glioblastoma who underwent surgical treatment; (3) described clinical or paraclinical characteristics relevant to neurorehabilitation (e.g., neurological deficits, cognitive impairment, functional status, imaging correlates, treatment-related complications) and/or reported rehabilitation interventions and associated functional outcomes; and (4) were published in English as full-text articles (“open-access”) in ISI-indexed journals [21], from 1 January 2022 to 31 December 2024.

Abstracts from conferences, case reports, publications that are not in the English language, studies lacking sufficient clinical and/or functional information, and studies with no accessible full text were excluded.

### 2.4. Study Quality Appraisal

**Figure 1:**
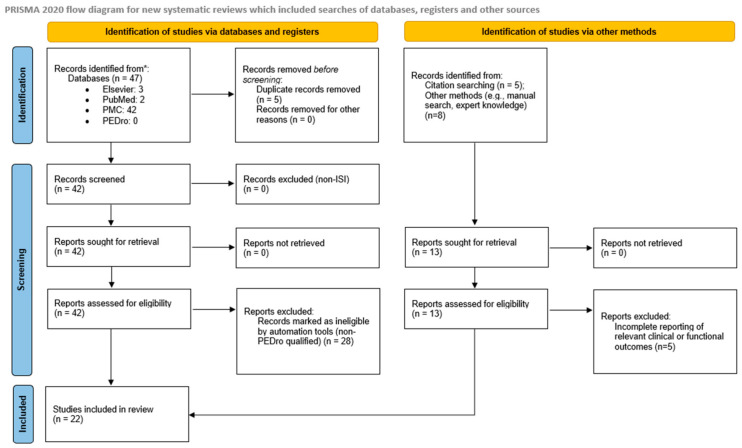
PRISMA type diagram [16] specific to our systematic literature review.

To assess the scientific impact of each paper, in addition to the PRISMA selection stages (Figure 1) and PRISMA checklist (Supplementary Material S1), we applied a qualitative selection based on the weighted average of citations per year, using a customized formula and relating to the PEDro scale (0–10 points) [22].

Thus, matching all previous filtering/selection PRISMA stages and also admitting a PEDro scale score of at least 4 (“fair quality” = PEDro score 4–5) [23], 14 articles remained relevant and were included in our systematic literature review and subjected to thorough analysis. The PEDro scale was selected as a pragmatic quality-screening tool because of the neurorehabilitation focus of the review. However, as several included studies were observational, review-based, bibliometric, or diagnostic in nature, PEDro scores should be interpreted with caution and not as a formal risk-of-bias assessment across all study designs.

To enhance the comprehensiveness of the present systematic review, manual reference screening of the 14 studies and complementary searches of specialized literature were performed. Any newly freely accessible identified articles were evaluated according to the same eligibility criteria (Appendix A). Thus, the final number of included articles was 22. The selection and the evaluation of individual articles were conducted concurrently by three of the authors, with any discrepancies resolved through discussion (Delphi type [24]) and consensus.

### 2.5. Advanced Quantitative Synthesis

Considering the heterogeneity of the studies and their specific effect sizes, the most defensible quantitative synthesis available from the extractable data is an exploratory pre-post meta-analysis of functional change on the Barthel Index (BI) scale [25]. Of the 22 included articles, only three datasets provided sufficiently comparable quantitative data on postoperative functional outcomes, reported on the BI scale, to permit exploratory quantitative synthesis. This analysis is clinically interpretable, but it remains non-randomized, largely single-arm, and potentially affected by cohort overlap between the two Natsume publications. The funnel plot generated should be used as a visual descriptive plot only, not as a formal test of publication bias.

(A) Extracted quantitative data

Table 2 depicts the eligible studies included in the quantitative synthesis.

(B) Statistical model

Effect measure: mean difference (MD) in BI points from postoperative/rehabilitation baseline to discharge/end of rehabilitation. An MD metric was retained because all extractable functional outcomes were reported on the same 0–100 BI scale.

Model: inverse-variance random-effects synthesis using the DerSimonian–Laird estimator for τ^2^. For each dataset, SE (change) was computed as sqrt[(SD0^2^ + SD1^2^ − 2rSD0SD1)/n], with r = 0.50 in the main analysis and r = 0.25/0.75 in sensitivity analyses. For further details, please see Table 3.

The pooled estimate was stable across plausible paired-correlation assumptions: approximately +31 BI points, with wider confidence intervals when lower pre-post correlation was assumed.

(C) Forest and Funnel plot

**Figure 2:**
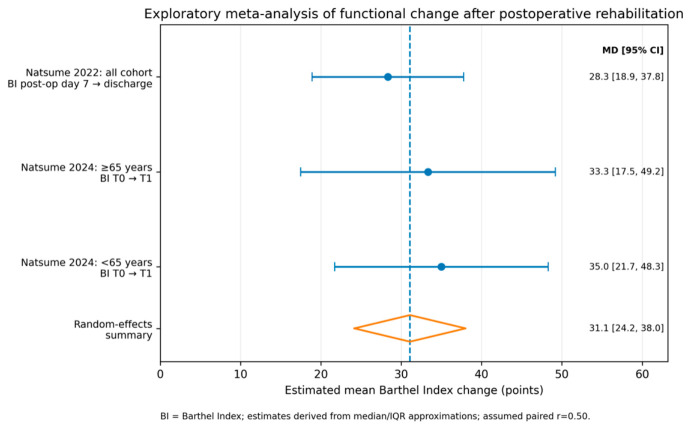
Exploratory inverse-variance meta-analysis of functional BI change after postoperative rehabilitation. The diamond represents the random-effects summary estimate. The dashed vertical line indicates the pooled mean Barthel Index change estimated by the random-effects model (31.1 points).

**Figure 3:**
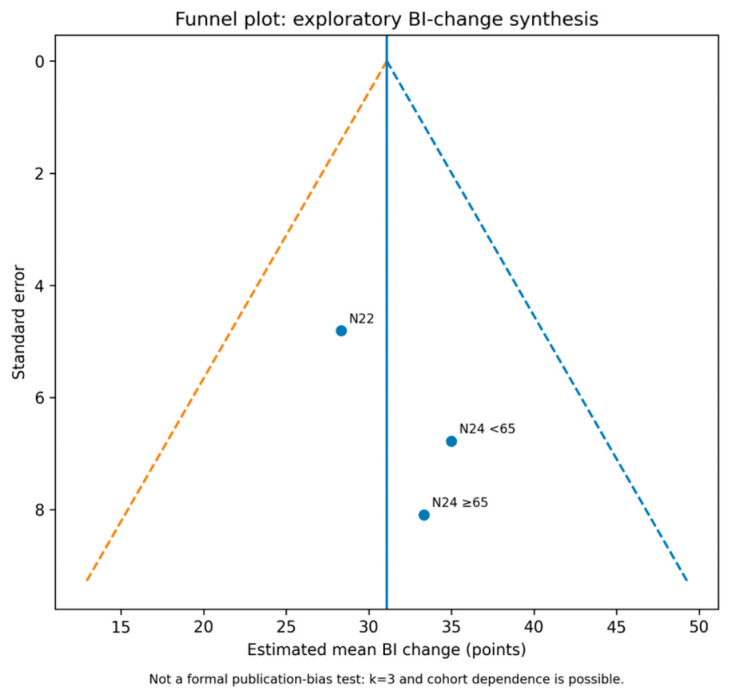
Funnel plot for the exploratory BI-change synthesis. The solid vertical line indicates the pooled mean BI change estimated by the random-effects model. The dashed oblique lines represent the approximate 95% pseudo-confidence limits of the funnel Because only three effect estimates were available and dependency between cohorts is possible, this plot must not be interpreted as a formal assessment of publication bias.

Forest and funnel plots (Figure 2 and Figure 3) were restricted to the exploratory BI-change synthesis. The plot did not permit a reliable inference regarding publication bias because only three effect estimates were available, and the Natsume datasets may involve overlapping patients. Accordingly, publication bias and small-study effects were assessed qualitatively rather than through Egger-type regression.

The exploratory synthesis indicates that there is a clinically meaningful improvement in the functional independence of patients in postoperative neurorehabilitation with a pooled estimate of an increase in BI score of 31.1 (95% CI 24.2 to 38.0). The direction of the effect was consistent across all extractable datasets. Nonetheless, this result should be interpreted as a descriptive functional-recovery signal rather than a causal estimate of rehabilitation efficacy, because the evidence base is observational, small, and not uniformly controlled. Thus, these findings should be considered exploratory and hypothesis-generating rather than confirmatory, considering the observational design and the limited number of works addressing rehabilitation interventions.

Quantitative synthesis was conducted only among studies reporting extractable outcomes of postoperative neurorehabilitation, measured on a common functional scale. Studies that reported non-quantitative, bibliometric, diagnostic, molecular, imaging, or non-neurorehabilitation outcomes were not included for quantitative synthesis. The primary continuous outcome was the change in the BI score from postoperative or rehabilitation baseline to discharge/end of rehabilitation. When BI values were reported as median and interquartile range, means and standard deviations were approximated using a quantile-based method consistent with Wan et al. [28]. The standard error of the difference was calculated under the assumption of a pre-post correlation of 0.50; sensitivity analyses were conducted for correlations of 0.25 and 0.75. We used an inverse-variance random-effects model, with DerSimonian–Laird estimation of between-study variance. A funnel plot was generated for visual appraisal of small-study effects; however, because fewer than ten effect estimates were available and overlapping cohorts could not be excluded, no formal funnel-plot asymmetry test was performed [28,29,30].

(D) Risk-of-bias/evidence-validity notes

Potential sources of bias and limitations affecting the internal validity of the included studies were systematically evaluated and are summarized in Table 4.

In order to maintain further rigor, this systematic review was prospectively registered in the International Prospective Register of Systematic Reviews (PROSPERO) Database under the number CRD420261324467.

## 3. Results

Fourteen studies were identified through the primary PRISMA search strategy (see Table 5). Subsequently, manual screening of reference lists yielded eight additional publications (see Appendix A) considered relevant to the objectives of the review, resulting in a final qualitative synthesis of 22 studies.

In order to enhance clarity while analyzing and interpreting the findings, the studies selected were initially grouped into three major categories (see Supplementary Material S2: Table S1): rehabilitation intervention and outcome studies (*n* = 7); neuro-oncology or supportive care studies relevant to rehabilitation (*n* = 8); and studies concerning biomarkers/imaging/molecular aspects (*n* = 7). The purpose of such classification was to separate the studies that examined rehabilitation interventions per se from those that provided context and indirect evidence relevant to rehabilitation and postoperative functional outcomes. Nevertheless, due to the multidisciplinary nature of the studied field, there were some overlaps among different topics, and many studies fit into more than one category; therefore, studies were classified according to their primary focus and main contribution to the review.

### 3.1. Genetic Predisposition

Brain and CNS tumors are highly heterogeneous. Most cases occur sporadically, although hereditary factors play an important role, particularly in high-grade gliomas [3]. Individuals with a familial history of glioma have an increased risk of developing this disease (approximately two-fold in those with first-degree relatives affected) [45]. This predisposition can be caused by constitutional defects in genes responsible for deoxyribonucleic acid (DNA) damage repair, maintenance of chromosomal stability, and regulation of cell cycle checkpoints [3,45]. There are many pathways that contribute to the susceptibility, but the main ones include tumor suppressor genes, most notably tumor protein p53 (TP53), which oversees programmed cell death and safeguards chromosomal integrity; Rat Sarcoma/Mitogen-Activated Protein Kinase (RAS/MAPK) signaling axis, whose dysregulation is closely tied to neurofibromatosis type 1 (NF1) loss-of-function variants; replicative polymerase proofreading fidelity, contingent upon DNA Polymerase Epsilon (POLE) and DNA polymerase delta 1 (POLD1) function; telomeric integrity and maintenance, governed in part by protection of telomeres. Disruption of these pathways leads to DNA damage accumulation, more mutations, and chromosomal instability, which are typical of malignant gliomas [3]. The absence of the mismatch repair pathway leads to a hypermutated phenotype in tumors; this phenomenon is common in high-grade gliomas, owing to inherent genetic predispositions [45]. Identifying these genetic alterations may facilitate better clinical management through genetic counseling, appropriate treatment, and surveillance [3].

### 3.2. Diagnosis and Monitoring

#### 3.2.1. Liquid Biopsy

Some authors suggest that analyzing cell-free DNA (cfDNA) from cerebrospinal fluid (CSF) is becoming increasingly important in the field of neuro-oncology, as it can support the diagnosis and molecular characterization of gliomas by revealing actionable genetic alterations. Compared with plasma, cfDNA is detected more frequently in CSF (up to 100% versus less than 10%), although plasma sampling remains a less invasive option for disease monitoring. Liquid biopsy can be used for diagnosis, to reflect tumor diversity, detect mutation and molecular profiling, particularly in cases with extensive disease or lesions located near the ventricles, when surgery is not possible. Additionally, extracellular vesicles released by glioblastoma cells and isolated from CSF are promising biomarkers, as they reflect tumor dynamics, carry specific molecular markers (e.g., Epidermal growth factor receptor variant III—EGFR v III), and provide prognostic information [35].

#### 3.2.2. Coronavirus Disease 2019 (COVID-19) and Delays in Diagnosis

A retrospective study that evaluated the effect of COVID-19 on neuro-oncology care found delays in the diagnosis and treatment of aggressive tumors like glioblastomas and brain metastases, for which prompt intervention is needed to avert the progression of the condition [46].

### 3.3. Glioblastoma Management

#### 3.3.1. Surgical Treatment

Standard surgical management of highly aggressive tumors such as glioblastoma involves cytoreductive surgery aiming for maximal resection of the contrast-enhancing tumor on magnetic resonance imaging (MRI) [14,42]. Complete resection is associated with improved progression-free and overall survival compared with partial excision. Nevertheless, in selected cases, subtotal resection may still yield favorable outcomes with a comparable level of risk [14].

Previous studies on high-grade glioma reported that repeated surgeries, despite their intrinsic risks and potential side effects, are still a safe and effective strategy for improving overall survival while striving to preserve QoL [41].

Clinical outcomes are influenced by several factors, including the extent of resection, tumor size and location, patient age, and molecular profile. In general, incomplete resection, larger or midline-crossing tumors, and unfavorable genetic characteristics are associated with a poorer prognosis [14].

Some techniques are used to maximize the extent of resection while preserving the function of normal brain structures. Among the most commonly used, we mention the following: intraoperative 5-aminolevulinic acid (5-ALA) guidance, MRI-based neuro-navigation, and awake surgery with intraoperative mapping [41,42]. Glioblastoma is also characterized by a high rate of local recurrence, which contributes to its poor prognosis. According to a retrospective, single-center study, C-methionine positron emission tomography (MET-PET) is a valuable neuro-oncological tool that can more precisely delineate the boundary between intracranial tumors and normal brain tissue than CT or MRI. This is useful in surgical planning, particularly for tumors located near eloquent areas. However, aggressive resection strategies must be balanced against the risk of postoperative neurological deficits and other complications [42].

Postoperative complications in patients undergoing brain tumor surgery may include ischemia, hemorrhage, infections, seizures, venous thromboembolic events, impaired wound healing, and hydrocephalus [32]. These may lead to a broad range of neurological and cognitive deficits, including motor weakness, sensory and visual disturbances, aphasia, dysphagia, ataxia, and/or cognitive impairment, often compounded by deconditioning and psychological stress [14].

Neurological deficits following brain tumor surgery may be temporary or permanent and can negatively impact functional status and prognosis. Motor deficits frequently improve during the months after surgery; cognitive impairments—particularly those affecting memory, executive function, and processing speed—tend to recover more slowly and often only partially. There are also frequent psychological complications related to surgery, such as anxiety and depression, that can negatively impact QoL, functional recovery, and survival. Tumor characteristics and adjuvant treatments, particularly whole-brain radiotherapy, may further increase these risks. To reduce surgical-related complications, structured perioperative functional assessment and brain mapping can be used, although standardized evaluation tools remain limited [14,32].

Following surgery, standard care for malignant brain tumors included radiotherapy and/or adjuvant chemotherapy, tailored to the patient’s clinical status as assessed by the Karnofsky Performance Scale (KPS) [46].

#### 3.3.2. Oncological Treatment

##### Radiotherapy

Radiotherapy is a key component in the treatment of infiltrative brain tumors, especially gliomas, which are seldom entirely resectable, being delivered with advanced techniques to maximize malignancy control while limiting damage to normal tissue [14,32]. It can be administered as whole-brain irradiation, fractionated therapy, or stereotactic radiosurgery. Although it is an essential part of the treatment, radiotherapy may be associated with cognitive decline and other adverse effects [14].

The risk increases with higher fraction doses (>2 Gy), greater total radiation dose, larger volumes of brain tissue being treated, and longer duration of treatment. Side effects occur in distinct phases: acute effects (such as headaches, fatigue, skin reactions, edema, and seizures), early delayed effects (such as demyelination and drowsiness), and late effects such as persistent cognitive impairment and radiation necrosis [14,32].

Long-term toxicity related to radiotherapy is also often associated with complex neurocognitive and psychiatric impairments such as memory loss, reduced attention span, slowed information processing, and motivational decline (particularly when the hippocampus is exposed to radiation). Additional neurological complications may include motor signs, especially gait disturbances, and urinary dysfunction, all of which can significantly affect QoL [14].

##### Chemotherapy

Chemotherapy in glioblastoma primarily includes TMZ, as part of the Stupp Protocol, which is an oral alkylating agent capable of crossing the blood–brain barrier and inducing cancer cell death through DNA damage, with treatment response influenced by MGMT status. Other agents used in selected settings are Lomustine and Bevacizumab [32].

Chemotherapy improves survival in patients with malignant brain tumors but is frequently associated with adverse reactions [14]. TMZ is associated mainly with hematological toxicity (e.g., thrombocytopenia, neutropenia), fatigue, and gastrointestinal symptoms. Lomustine may cause delayed myelosuppression and Bevacizumab is linked to hypertension, thromboembolic events, impaired wound healing, and increased risk of bleeding. Chemotherapy also may impair immune response and increase the risk of infections, including viral reactivation [32].

Another important adverse effect is chemotherapy-induced cognitive dysfunction (CICD), which affects more than 30% of glioma patients. These effects may be difficult to distinguish from those caused by radiotherapy. Risk depends on treatment intensity, dose, duration, and patient-related factors, including genetics, education, and neurological history. Some of the common cognitive deficits include impairments in memory, attention span, information-processing speed, language, and executive functions. Potential causes include neurotoxicity, white matter and/or vascular injury, oxidative stress, and impaired neurogenesis. These impairments may reduce independence, work capacity, and social participation. Rehabilitation strategies combining cognitive training, physical activity, and psychological support may help alleviate symptoms and improve overall functioning and QoL [14].

##### Targeted Treatment, Including IDH-Specific and Virotherapy

A bibliometric analysis of the literature highlights oncolytic virotherapy (which uses engineered viruses to selectively destroy tumor cells and stimulate anti-tumor immune responses) as a promising therapeutic strategy for glioblastoma. More precisely, a phase II trial in Japan showed promising results with triple-mutated, third-generation oncolytic herpes simplex virus type 1 (G47Δ) in recurrent glioblastoma, leading to its approval in 2021 as the first oncolytic virus for malignant glioma [33]. Another bibliometric study has shown that glioma stem cells (GSCs) are key therapeutic targets driving treatment resistance (such as TMZ—noncompliance). In this context, epithelial-to-mesenchymal transition (EMT) is a cellular process essential for embryonic development that also plays a key role in cancer progression by promoting tumorigenesis, invasion, metastasis, and resistance to chemo- and radio-therapy. Additionally, in tumors, EMT involves the dynamic transformation of epithelial cells into mesenchymal ones, enabling cancer cells to gain stem-like properties, enhanced motility, and invasiveness, and moreover, in gliomas, EMT further facilitates immune evasion, immunosuppression, and resistance to current treatments [44].

In addition, epigenetic biomarkers play a key role in altered gene expression and in the pathogenesis of numerous human cancers. Specifically, an in vitro study investigates the correlation between Long Intergenic Non-Protein Coding RNA 2587 (LINC02587) expression and the clinical-pathological characteristics of glioma patients. The results demonstrated that silencing LINC02587 reduced proliferation, migration, and invasion of glioma cells while increasing apoptosis, suggesting its potential as a novel biomarker in this type of malignancy. On the other hand, epigenetic modifications, particularly DNA methylation, are widely considered to govern tumor development and the occurrence of secondary malignant growth [40].

These complex and aggressive pathways help explain why, although therapeutic strategies are continuously advancing, survival rates in glioma—particularly glioblastoma—remain very poor.

##### Future Perspectives in Neuro-Oncology

Emerging developments in neuro-oncology are increasingly linked to the integration of digital health technologies, especially artificial intelligence (AI) and extended reality (XR). The integration of clinical, imaging, and molecular data may improve diagnostic accuracy, prognostic assessment, and treatment planning. At the same time, XR technologies are beginning to be recognized as valuable in surgical planning, training, and patient involvement. They have become a modern facility used in neurorehabilitation. However, significant challenges remain, including issues related to data reliability, standardization, clinical validity, and real-world implementation. As such, the need for sustained research and interdisciplinary collaboration must be emphasized [34].

#### 3.3.3. Complications Relevant to NeuroRehabilitation

The European Association of Neuro-Oncology (EANO) has issued comprehensive guidelines in a study aimed at the prevention, identification, and management of adverse effects and complications in adults with primary CNS malignant tumors, with particular emphasis on surgical, radiotherapeutic, and pharmacological treatments [32].

Perilesional edema may also occur following surgery or radiotherapy. It can contribute to temporary or persistent neurological and cognitive deterioration, mainly due to increased intracranial pressure and blood–brain barrier disruption. Edema that develops after surgery can be associated with short-term impairments in memory, attention, language, and executive function. In contrast, radiation-induced edema usually develops within months and may lead to a more sustained decline in cognitive performance [14].

Seizures are a common complication in brain tumor patients, notably in glioblastoma. They may occur during surgery or can be caused by tumor extension. Despite this, prophylactic antiepileptic treatment is not routinely recommended. These episodes are associated with tumor-induced brain irritation and elevated intracranial pressure. They adversely impact cognitive function and overall QoL [14,32].

Fatigue is a frequent complication of systemic manifestations of malignancy as well as of radiotherapy and chemotherapy, affecting more than half of patients. It reduces cognitive efficiency, functional capacity, and work performance, and is associated with depression, reduced alertness, and increased risk of secondary complications such as falls and prolonged hospitalization [14].

Psychocognitive impairment is particularly important, especially in cases involving the dominant hemisphere or frontal lobe, rapid tumor growth, and extensive resection of large, deep gliomas affecting cortical and subcortical white matter. Anxiety and depression, frequently observed in these patients, can additionally contribute to the clinical picture and negatively impact QoL [14,37]. Factors associated with cognitive decline may include tumor location, mass effect, edema, epilepsy, medication use, and older age. On the other hand, chemo- and radio-therapy appear less predictive [14]. Pharmacological and non-pharmacological interventions, including cognitive rehabilitation, physical and occupational therapy, virtual reality training, and hyperbaric oxygen therapy, have shown potential benefits [37].

Motor impairment may arise from tumor involvement of motor pathways (during disease progression) and/or as a consequence of treatment (mainly occurring postoperatively) and may be transient or permanent. Importantly, motor deficits contribute to reduced independence, lower functional capacity, and poorer QoL, and may increase the risk of secondary complications [32]. Their severity depends on tumor location, edema, surgical effects, disease progression, and psychological factors. Although perioperative and rehabilitation assessments rely on different instruments, functional scales generally provide a better estimate of long-term independence. However, no standardized assessment system exists, highlighting the need for integrated multidisciplinary evaluation [14].

Swallowing and speech-language deficits

Dysphagia is a relatively common complication in patients with high-grade gliomas, with reported prevalence ranging from 8% to 79%. It may result from sensory-motor (especially when related cranial nerves are affected) and/or cognitive impairments related to tumor progression, treatment effects, or a combination of both. All phases of swallowing can be affected—oral, pharyngeal, and esophageal [15].

Language deficits are often associated with brain tumors, especially malignant ones, with greater invasiveness, affecting dominant-hemisphere language networks and major white matter pathways [14].

### 3.4. Treatment Goals

Patients with glioblastoma represent a unique challenge for clinicians, including limited life expectancy, rapid neurological, psychocognitive, and/or emotional decline [13]. Given the higher grade and aggressiveness of glioblastoma, it has an even greater negative impact on health-related QoL, particularly affecting survival, respectively, physical and social functioning [39].

#### 3.4.1. Oncological Goals

Pre- and postoperative tumor volumes are key predictors of survival, with smaller residual volumes linked to better outcomes. Despite this, current guidelines still rely on simplified risk categories and may overlook volumetric and molecular factors, leading clinicians to sometimes postpone adjuvant therapy in order to minimize long-term treatment toxicity [36].

#### 3.4.2. Onco-Functional Balance

The primary challenge in the management of gliomas is reaching the optimal balance between maximal tumor resection and the preservation of neurological and cognitive functions [36,47]. This concept of “onco-functional balance” guides not only surgical choices, particularly when tumors are located near eloquent brain regions, but also extends to the planning of radiotherapy and chemotherapy. The goal is to maximize tumor control while minimizing functional impairment. It also emphasizes neuroplasticity and supports personalized care, taking into account factors specific to each patient [36].

#### 3.4.3. Functional Goals

Patient performance and functional status are usually assessed using the KPS, a standard tool in neuro-oncology [23,46].

The natural progression of the disease, along with the cumulative adverse effects of oncological treatments, including surgery, can significantly impair patients’ functionality and related QoL. These impacts extend across autonomy, cognitive performance, professional functioning, and overall well-being, and may occur early in the disease course—even prior to treatment—as well as during later stages of management. As a result, long-term preservation of QoL has become a central objective in oncological care. Health-related QoL is influenced by a complex interplay between functional independence and behavioral, neuropsychological, and social factors that may undermine it. Therefore, two clinical outcomes can be identified as the “tip of the iceberg”: cognitive performance (tracked through repeated assessments over time) and work reintegration, which indicates both the patients’ ability to perform complex functioning over a sustained period of time, and the broader socio-economic effects of treatment [36].

### 3.5. Effects on Patients’ QoL—The Contribution of NeuroRehabilitation

Neurorehabilitation is essential in both benign and malignant brain tumors and should be part of all treatment plans [14].

The primary goal of neurorehabilitation is to maximize functional outcomes. Additional objectives such as symptom management, autonomy enhancement, and QoL improvement are also taken into account [13,14].

In a study of glioblastoma patients, ADLs were assessed using the BI (scores range from 0 to 100), at both the start of postoperative rehabilitation and at discharge. Higher BI values reflected greater independence. Moreover, the BI score at discharge was demonstrated to be a key predictor of improved survival outcomes [27].

A separate study by the same authors has shown that preoperative baseline BI values influenced functionality during hospitalization, allowing the patients to be classified into deterioration and good recovery groups. Moreover, after undergoing a rehabilitation program, the mean BI score was significantly improved [26].

A multidisciplinary/multiprofessional team approach, including physical and rehabilitation doctors and physio-/kinesio-therapists, speech and language and/or swallowing therapists, occupational therapists, and social workers, is required [13,43].

Although patients with glioblastoma frequently present with poor preoperative functional status, a structured early inpatient rehabilitation intervention may improve postoperative functional outcomes [26].

Inpatient rehabilitation has been shown to have considerable effectiveness in individuals with brain tumors, with functional improvements and discharge rates similar to those observed in patients with other neurologic conditions, such as stroke or traumatic brain injury. A common therapeutic pathway of neurorehabilitation is to promote appropriate adaptive (opposite to maladaptive) neuroplasticity (see below) [48]. Typically, rehabilitation includes physical therapy, rehabilitative nursing care, speech and swallowing therapy—when needed, occupational therapy, cognitive training, and psychological and health educational support for patients with glioma and their families/caregivers. The timing of rehabilitation varies among individuals, ranging from the early postoperative period during hospitalization to weeks after active medical management, and it can be conducted in inpatient, outpatient, or home-based settings [13].

Physical therapy in glioblastoma patients offers multiple benefits, focusing on improving mobility, strength, and overall physical—including even survival—and psychological well-being, which may support self-autonomy, QoL [13,14].

Immediate postoperative care includes supportive general care, encompasses rehabilitation nursing, which consists of close monitoring of vital signs, pain management, appropriate nutritional support, head elevation, frequent repositioning, and respiratory exercises to prevent complications and facilitate recovery. The process of progressive mobilization, gradual limb exercises, and short periods of sitting is initiated on the first day after surgery. The degree of activity has to be tailored to each patient’s tolerance level and progressively increases accordingly. Muscle strength is constantly evaluated to guide progression from bedside sitting to standing, then assisted walking, and eventually independent ambulation [27,43].

Thus, neurorehabilitation helps address common issues such as gait and balance problems, muscle weakness, and fatigue, often worsened by disease and its respective treatments. Interventions generally include individualized gait training—supported by advanced assistive devices, balance work, resistance and aerobic exercise, and multicomponent programs that are both safe and feasible even during active treatment [13].

Exercise-based and structured rehabilitation techniques improve physical capacity and support cognitive and psychological recovery [14]. Several studies have reported a reduction in cancer-related fatigue following aerobic exercise. More precisely, they suggested that early walking training may be important not only for the cancer-related fatigue but also for improving the functional and/or mental status of patients with glioblastoma [14,26,27]. Physical therapy may also play a role in prehabilitation by addressing pain—when present, enhancing physical fitness before surgery and/or chemo- and/or radio-therapy, and improving patients’ ability to tolerate medical stress while potentially mitigating treatment-related side effects. Overall, exercise-based rehabilitation should be personalized to patient needs and preferences to maximize adherence and functional outcomes [13,14].

Comprehensive swallowing and speech-language therapy is essential for evaluation, diagnosis, and individualized management, including patient and caregiver education. As noted above, dysphagia is a frequent complication in glioblastoma patients, with severe consequences such as aspiration pneumonia, malnutrition, dehydration, medication noncompliance, reduced QoL, and increased mortality [15]. In addition, malignant brain tumors and their treatments may result in speech disorders that impair communication and consequently, QoL [14]. Despite their high prevalence and clinical impact, both dysphagia and speech-language disorders remain underdiagnosed and undertreated in patients with glioblastoma [15], possibly because of their frequently high aggressiveness, with multiplan biofunctional severe impairments, which have forced prioritized focus on life-saving interventions.

Occupational therapy provides support for patients with glioblastoma by improving daily functioning, independence, and participation in meaningful daily activities, and also promoting social/professional engagement and enhancing overall QoL [13,14]. Evidence from retrospective and randomized studies indicates that it may enhance functional and cognitive abilities and help reduce fatigue, especially during acute treatment and active phases of care [14]. Therapists can apply both restorative and compensatory strategies, and may involve family members and caregivers. These strategies include the use of dedicated assistive devices, training in ADL, fatigue management, routine structuring, and education on sleep hygiene and symptom control. As part of a multidisciplinary/multiprofessional team, occupational therapy helps address functional impairments (e.g., mobility, cognition, upper limb function, etc.) and thereby enhances QoL, although more research is needed to define optimal-related protocols and outcomes [13].

Cognitive rehabilitation provides support for patients aiming to address frequent impairments in attention, memory, perception and executive function, resulting both from the tumor and its treatments. Given that cognitive status may fluctuate over the course of the disease, individualized ongoing assessment is essential in order to dynamically adapt therapeutic strategies and means.

These interventions can include pharmacological and/or non-pharmacological components and may promote neuroplasticity and functional recovery through cognitive training, goal management and specific exercises. Neuropsychological evaluation is currently regarded as the gold standard for detecting impairment, developing a treatment plan, and suggesting compensatory strategies for ADLs, occupational tasks, and decision-making. Overall, cognitive rehabilitation promotes adaptation and independence and leads to improved QoL, but requires ongoing monitoring [13,14].

A personalized, multidisciplinary/multiprofessional approach is needed in order to enhance functional recovery, participation, and QoL. To achieve those, it is necessary to appropriately integrate clinical assessments and patient-reported outcomes within a holistic framework [14].

### 3.6. Interventional NeuroRehabilitation and Brain Plasticity

A narrative review—particularly valuable for highlighting differences compared with glioblastoma characteristics—outlines clinical, radiological, and oncological markers that reflect the brain’s plastic potential in diffuse low-grade glioma (LGG) management and examines how neural plasticity interacts with both the natural course of the disease and therapeutic interventions. It proposes a multimodal, staged treatment strategy that incorporates individualized brain plasticity profiling to guide personalized decision-making [36].

In a proof-of-concept study, the safety of “interventional neurorehabilitation”, a personalized approach using connectome-based TMS, to promote cortical reorganization after craniotomy, was assessed. Glioma patients with postoperative motor or language deficits received targeted stimulation based on individual brain network mapping. The strategy aimed to enhance neuroplasticity and support early functional recovery, especially when surgical interventions impacted affected critical brain regions. The study demonstrated that, in a cohort of 31 patients who underwent glioblastoma surgery, the administration of rTMS in conjunction with standard rehabilitation approaches/strategies enhanced motor and language deficits, as evaluated on specific clinical scales [31].

Another review reports on how early surgical intervention and gross-total or supratotal resection reduce the risk of infiltration and are well substantiated in prolonging overall survival in lower- and higher-grade gliomas. Although awake craniotomy with brain mapping remains the gold standard for tumors located near eloquent cortex, neuromodulation-induced cortical prehabilitation (NICP)—employing techniques such as rTMS or extraoperative direct cortical stimulation (eDCS)—is being investigated as a noninvasive alternative [38].

## 4. Discussion

Considering glioblastoma as one of the most aggressive tumors of the CNS [4,5,6], early diagnosis and appropriate therapeutic management are essential to improve survival and to counteract, as much as possible, tumor recurrence, disease progression, associated disabilities, overall decline in patients’ QoL, and, not least, the mortality burden spectrum it brings [6].

Patients with brain tumors often experience neurological, cognitive, and emotional symptoms that become more pronounced with disease progression or recurrence. Common issues include seizures, cognitive decline, fatigue, pain, and mood disturbances, as well as anxiety and/or depression, which impede independence, social interactions, and work capacity. Overall biological stamina, functional status, cognitive ability, and symptom burden are key determinants of survival and QoL, highlighting the need for early, multidisciplinary/multiprofessional, patient-centered care supported by comprehensive rehabilitation, as well as psychological, social, and community support [4].

Modern neuro-oncology care promotes early, integrated, patient-centered management involving multiple professionals from diagnosis onward [25].

Glioma-related somatic symptoms and cognitive function can change over time due to tumor progression and therapeutic interventions, leading to fluctuating or persistent neurobiopsychological deficits. As previously emphasized, despite exhaustive therapeutic and rehabilitative interventions, outcomes remain variable: some patients experience meaningful functional gains, while others continue to decline [14].

In this complex interaction, patients may develop complications arising both from the tumor itself and from the aggressiveness of antitumor treatment, leading to neuromotor and/or psycho-cognitive decline, as well as an overall decrease in functionality and QoL, in the case of survival [47,49].

In clinical practice, a multidisciplinary and multiprofessional team of healthcare professionals, including surgeons, oncologists, rehabilitation physicians, palliative care specialists, and supportive care therapists, provide coordinated and continuous care throughout hospitalization to address the biological, psychological, and social needs of patients and optimize clinical and functional outcomes. Within this framework, neurorehabilitation should be considered an essential part of the comprehensive therapeutic management for these patients [14]. Supportive general care, including rehabilitative nursing and comprehensive neurorehabilitation programs encompassing physical, occupational, and speech/swallowing therapy, as well as psychosocial and (where the case demands) palliative care—should all be provided during hospitalization and continued after discharge [14,43]. The implications of both healthcare professionals, but also the patient’s caregivers improve adherence, provide emotional support, and enhance overall effectiveness [14].

Applying the principles of P4—precision medicine (predictive, preventive, personalized, and participatory) in this context, may help shift the focus from purely survival-based metrics toward anticipatory functional assessments, risk-stratified interventions, and active patient participation throughout the disease course [50,51].

Available evidence has shown that inpatient rehabilitation may play a valuable role in increasing functional independence, can improve ADL in individuals affected by brain tumors, with comparable benefits to those observed in patients recovering, within rehabilitation, from stroke or traumatic brain injury, although it is not as extensively studied. However, most research includes heterogeneous brain tumor populations, while relatively few studies specifically examine patients with glioblastoma. Rehabilitation interventions for individuals with gliomas have been suggested to be feasible, well-tolerated, safe, and capable of improving disability and QoL [13,14,27].

Despite these findings, the influence of rehabilitation on functional outcomes and survival, especially among older patients with glioblastoma, remains underexplored [27].

While conducting this systematic literature review, we observed that due to the limited number of articles on this topic, the chosen subject—neurorehabilitation in surgically treated glioblastomas—is insufficiently explored within the current bibliographic resources. The findings of the present PRISMA systematic literature review, enriched as a knowledge base by supplementary freely available bibliographic resources, provide valuable insights, indicating that advances in tumor markers, diagnostic imaging, surgical techniques and technologies, and, equally important, neurorehabilitation interventions (including nursing/care-related type) and adjuvant therapies have collectively contributed to a relative increase in survival rates and improvements in clinical condition and QoL among patients with glioblastoma. Our exploratory pre-post meta-analysis of BI change demonstrated a pooled improvement of approximately 31 points from rehabilitation baseline to discharge, suggesting substantial functional gains during the postoperative rehabilitation period [25]. Our results are supported by the existing neurorehabilitation literature, which has shown improvements in functional autonomy following rehabilitation interventions [13,14]. However, because the analysis was based on a limited number of heterogeneous, predominantly single-arm studies, the pooled estimate should be interpreted as exploratory evidence describing recovery trajectories rather than as definitive evidence of treatment efficacy.

**Limitations**

This systematic review has several limitations that should be considered. Firstly, the included articles are heterogeneous, considering the types of approach used (from diagnosis to multimodal interventions), the timing, and the setting of each work. This may limit any direct comparisons among studies. Secondly, the measurement of outcomes may vary across studies, limiting their comparability. Thirdly, most of the works included here are single-arm and small-scale retrospective investigations, having an inherent risk of bias. All these limitations suggest that the evidence presented in our review is exploratory. Furthermore, in patients with glioblastoma, functional evolution depends on tumor progression and its oncological management. Thus, the specific effect of rehabilitation is difficult to isolate. In addition, evidence for newer approaches, such as neuromodulation-based [38,52] interventions, remains preliminary. Consequently, this review should be regarded as hypothesis-generating and intended to identify promising directions for future research rather than to provide definitive evidence regarding rehabilitation efficacy.

## 5. Conclusions

Despite extensive research and thoroughly deployed scientific endeavors (particularly over the past decades), progress in combating this devastating disease remains below expectations. Under these circumstances, continued determination in advancing research—including the expansion of available therapeutic interventions—remains an obvious priority. Given the substantial functional deficits associated with glioblastoma and its treatment, early referral to rehabilitation services is essential for optimizing recovery and maintaining patient independence. This aspect is highlighted in our exploratory pre-post meta-analysis that showed an average pooled increase in BI points from baseline rehabilitation assessment to discharge.

In this regard, we consider that until an effective cure for glioblastoma can be found, neurorehabilitation offers a contributive type of medical intervention that warrants continuous development and further rigorous investigation. Thus, this topic has only been partially explored in recent years, offering opportunities for both, theoretical and practical, in-depth investigation, as well as potential original contributions.

## Figures and Tables

**Table 1:** Contextually keyword combinations/“syntaxes” used for searching of bibliographic resources in four international medical databases.

Keywords	Elsevier	PubMed	PMC	PEDro	Total
“Neurorehabilitation” and “Glioblastoma” and “Neurosurgery” and “Neuro-oncology”	3	2	37	0	42
“Neurorehabilitation” and “Recovery” and “Neuro-oncology” and “MGMT” and “IDH mutation” and “IDH wilde type” + “Biomarkers”	0	0	0	0	0
“Neurorehabilitation” and “Targeted therapies” and “Temozolomide” and “Recovery”	0	0	5	0	5
Total	3	2	42	0	47

**Table 2:** Results of the extracted quantitative data. Values are median [Interquartile Range (IQR)] as reported by the primary articles. Means and standard deviations were approximated from median/IQR summaries using a Wan-type quantile approach. The primary paired-correlation assumption was r = 0.50. Depicts the eligible studies included in the quantitative synthesis.

Study	Dataset	*n*	BI Baseline	BI Follow-Up	Estimated MD	SE	Comment
Natsume et al., 2022 [26]	All cohort; postoperative nadir to discharge	105	7 days after surgery: 30 (0–60)	Discharge: 65 (20–90)	28.3	4.81	Same institution/timeframe as Natsume 2024; possible cohort overlap. Used as exploratory complementary dataset.
Natsume et al., 2024 [27]	Older subgroup (≥65 years)	45	T0, beginning of rehabilitation: 30 (0–55)	T1, end of rehabilitation: 75 (15–95)	33.3	8.09	Age-stratified subgroup; same parent study as younger subgroup.
Natsume et al., 2024 [27]	Younger subgroup (<65 years)	30	T0, beginning of rehabilitation: 35 (15–65)	T1, end of rehabilitation: 75 (50–95)	35.0	6.78	Age-stratified subgroup; same parent study as older subgroup.

**Table 3:** Summary of exploratory meta-analysis of BI change and sensitivity to assumed pre-post correlation.

**k**	**Pooled MD**		**95% CI**		**τ^2^**	**Q**	**df**	**I^2^**
3	31.09		24.17 to 38.01		0.000	0.74	2	0.0%
**Assumed r**		**Pooled MD**		**95% CI**		**τ^2^**	**I^2^**	
0.25		31.10		22.69 to 39.52		0.000	0.0%	
0.50		31.09		24.17 to 38.01		0.000	0.0%	
0.75		31.07		26.08 to 36.05		0.000	0.0%	

**Table 4:** Results of the risk-of-bias assessment for the included studies.

Study	Main Concern	Judgement
Natsume et al. 2022 [26]	Retrospective single-center cohort; no non-rehabilitation control; outcome-defined groups; possible overlap with 2024 cohort.	Moderate/high
Natsume et al. 2024 [27]	Retrospective single-center cohort; age comparison rather than no-rehabilitation comparator; same institutional timeframe as 2022 publication.	Moderate/high
Poologaindran et al. 2022 [31]	Proof-of-concept rTMS study; uncontrolled; open-label; short follow-up; valuable safety/feasibility signal but not directly poolable with BI rehabilitation outcomes.	High for efficacy, lower concern for acute safety reporting

**Table 5:** Table with authors, titles, and journals of selected articles in our systematic literature review.

No.	Article	Pub. Year	Citations Count	PEDro Score
1.	Michael Weller et al., Diagnosis and management of complications from the treatment of primary central nervous system tumors in adults, Neuro Oncol. 2023 Jul; 25(7): 1200–1224. Published online 27 February 2023. doi: 10.1093/neuonc/noad038 [32].	2023	10	10
2.	Anujan Poologaindran et al., Interventional neurorehabilitation for promoting functional recovery post-craniotomy: a proof-of-concept, Sci Rep. 2022; 12: 3039. Published online 23 February 2022. doi: 10.1038/s41598-022-06766-8 [31].	2022	7.333333	10
3.	Kunming Cheng et al., Emerging trends and research foci of oncolytic virotherapy for central nervous system tumors: A bibliometric study, Front Immunol. 2022; 13: 975695. Published online 6 September 2022. doi: 10.3389/fimmu.2022.975695 [33].	2022	8.333333	10
4.	Tawseef Ayoub Shaikh, Tabasum Rasool Dar, Shabir Sofi, A data-centric artificial intelligent and extended reality technology in smart healthcare systems, Soc Netw Anal Min. 2022; 12(1): 122. Published online 1 September 2022. doi: 10.1007/s13278-022-00888-7 [34].	2022	6.333333	10
5.	Giulia Berzero et al., The coming of age of liquid biopsy in neuro-oncology. Brain. 2023 Oct; 146(10): 4015–4024. Published online 8 June 2023. doi: 10.1093/brain/awad195 [35].	2023	4.5	8
6.	Sam Ng, Hugues Duffau, Brain Plasticity Profiling as a Key Support to Therapeutic Decision-Making in Low-Grade Glioma Oncological Strategies, Cancers (Basel) 2023 Jul; 15(14): 3698. Published online 20 July 2023. doi: 10.3390/cancers15143698 [36].	2023	4.5	8
7.	Matthew A et al., A systematic review of cognitive interventions for adult patients with brain tumours, Cancer Med. 2023 May; 12(10): 11191–11210. Published online 7 March 2023. doi: 10.1002/cam4.5760 [37].	2023	4	7
8.	Ryan P. Hamer, Tseng Tsai Yeo, Current Status of Neuromodulation-Induced Cortical Prehabilitation and Considerations for Treatment Pathways in Lower-Grade Glioma Surgery, Life (Basel) 2022 Apr; 12(4): 466. Published online 22 March 2022. doi: 10.3390/life12040466 [38].	2022	4	7
9.	Sé Maria Frances et al., Long-term health-related quality of life in meningioma survivors: A mixed-methods systematic review, Neurooncol Adv. 2024 Jan–Dec; 6(1): vdae007. Published online 19 January 2024. doi: 10.1093/noajnl/vdae007 [39].	2024	4	7
10.	Zhengang Wang et al., DNA methylation-regulated LINC02587 inhibits ferroptosis and promotes the progression of glioma cells through the CoQ-FSP1 pathway, BMC Cancer. 2023; 23: 989. Published online 17 October 2023. doi: 10.1186/s12885-023-11502-0 [40].	2023	3	6
11.	Hugues Duffau, Repeated Awake Surgical Resection(s) for Recurrent Diffuse Low-Grade Gliomas: Why, When, and How to Reoperate? Front Oncol. 2022; 12: 947933. Published online 5 July 2022. doi: 10.3389/fonc.2022.947933 [41].	2022	3.333333	6
12.	Kazufumi Ohmura et al., Resection of positive tissue on methionine-PET is associated with improved survival in glioblastomas, Brain Behav. December 2023; 13(12): e3291. Published online 2023 Oct 16. doi: 10.1002/brb3.3291 [42].	2023	2.5	5
13.	Lydia Karamani et al., Tumor size, treatment patterns, and survival in neuro-oncology patients before and during the COVID-19 pandemic, Neurosurg Rev. 2023; 46(1): 226. Published online 6 September 2023. doi: 10.1007/s10143-023-02132-y [43].	2023	2	4
14.	Sirong Song et al., Global research trends and hotspots on glioma stem cells, Front Oncol. 2022; 12: 926025. Published online 29 September 2022. doi: 10.3389/fonc.2022.926025 [44].	2022	2.333333	4

## Data Availability

Not applicable.

## Supplementary Materials

The following supporting information can be downloaded at: https://www.mdpi.com/article/10.3390/life16071092/s1, Supplementary Materials: Supplementary Material S1: PRISMA checklist, Supplementary Material S2: Table S1. Classification of Included Studies According to Their Primary Focus. Reference [53] are cited in the supplementary materials.

## Abbreviations

The following abbreviations are used in this manuscript:

5-ALA	5-aminolevulinic acid
AI	artificial intelligence
BI	Barthel Index
CICD	chemotherapy-induced cognitive dysfunction
cfDNA	cell-free DNA
COVID-19	Coronavirus Disease 2019
CSF	cerebrospinal fluid
DNA	deoxyribonucleic acid
EANO	European Association of Neuro-Oncology
eDCS	extraoperative direct cortical stimulation
EGFR v III	Epidermal growth factor receptor variant III
EORTC	European Organization for Research and Treatment of Cancer
EMT	Epithelial-to-mesenchymal transition
GBM	Glioblastoma Multiforme
GSC	glioma stem cells
IDH	isocitrate dehydrogenase
IQR	Interquartile Range
KPS	Karnofsky Performance Scale
MET-PET	C-methionine positron emission tomography
LINC02587	Long Intergenic Non-Protein Coding RNA 2587
LGG	low-grade glioma
MD	mean difference
MGMT	Methylguanine-DNA Methyltransferase
MRI	Magnetic Resonance Imaging
NF1	neurofibromatosis type 1
NICP	neuromodulation-induced cortical prehabilitation
PEDro	Physiotherapy Evidence Database
POLD	polymerase delta 1
POLE	Polymerase Epsilon
PMC	PubMed Central
PRISMA	Preferred Reporting Items for Systematic Reviews and Meta-Analyses
PROSPERO	International Prospective Register of Systematic Reviews
QoL	Quality of life
RAS/MAPK	Rat Sarcoma/Mitogen-Activated Protein Kinase
rTMS	repetitive Transcranial Magnetic Stimulation
TMZ	Temozolomide
TP53	tumor protein p53
XR	extended reality
WHO	World Health Organization

## Appendix A. Supplementary Articles Included in Our Systematic Review

**No.**	**Article**	**Pub. Year**	**Citations Count**
1	Gambarin M, Malgrati T, Censo R Di et al. An Overview of Reviews on Predictors of Neurorehabilitation in Surgical or Non-Surgical Patients with Brain Tumours. Life 2024, Vol 14, Page 1377 2024;14(11):1377. https://doi.org/10.3390/LIFE14111377.	2024	5
2	Natsume K, Yoshida A, Sakakima H et al. Age-independent benefits of postoperative rehabilitation during chemoradiotherapy on functional outcomes and survival in patients with glioblastoma. Journal of Neuro-Oncology 2024 170:1 2024;170(1):129–37. https://doi.org/10.1007/S11060-024-04785-1.	2024	7
3	Zanotto A, Glover RN, Zanotto T et al. Rehabilitation in People Living with Glioblastoma: A Narrative Review of the Literature. Cancers 2024, Vol 16, Page 1699 2024;16(9):1699. https://doi.org/10.3390/CANCERS16091699.	2024	17
4	Natsume K, Sakakima H, Kawamura K et al. Factors Influencing the Improvement of Activities of Daily Living during Inpatient Rehabilitation in Newly Diagnosed Patients with Glioblastoma Multiforme. Journal of Clinical Medicine 2022, Vol 11, Page 417 2022;11(2):417. https://doi.org/10.3390/JCM11020417.	2022	11
5	Ostrom QT et al., 2022 Ostrom QT, Price M, Neff C, Cioffi G, Waite KA, Kruchko C, Barnholtz-Sloan JS. CBTRUS Statistical Report: Primary Brain and Other Central Nervous System Tumors Diagnosed in the United States in 2015-2019. Neuro Oncol. 2022 Oct 5;24(Suppl 5):v1–v95. doi: 10.1093/neuonc/noac202. PMID: 36196752; PMCID: PMC9533228.	2022	1708
6	Das A, Ercan AB, Tabori U. An update on central nervous system tumors in germline replication-repair deficiency syndromes. Neurooncol Adv 2024;6(1). https://doi.org/10.1093/NOAJNL/VDAE102.	2024	20
7	Bakare, Ajibola B., Aslam, Rizwan. A systematic review of dysphagia prevalence and the role of Otolaryngologists in high-grade glioma management. Journal of Laryngology and Voice 15(1):p 10–18, January–June 2025.|DOI: 10.4103/jlv.JLV_3_25.	2025	0
8	Ohmura K, Daimon T, Ikegame Y et al. Resection of positive tissue on methionine-PET is associated with improved survival in glioblastomas. *Brain Behav* 2023;13(12):e3291. https://doi.org/10.1002/brb3.3291.	2023	10
