# Supplementary material for: Clinical and Paraclinical Characteristics Relevant to NeuroRehabilitation and Their Outcomes in Postoperative Glioblastoma Patients: A PRISMA Systematic Literature Review
Source: Life (Basel). 2026 Jun 29;16(7):1092. doi: 10.3390/life16071092 (PMC13412169; doi:10.3390/life16071092)
Supplement: Supplementary file 1 [file life-16-01092-s001.zip › Supplementary Material S2.pdf]

Table S1. Classification of Included Studies According to Their Primary Focus

No.	Study	Rehabilitation-focused studies	Neuro-oncology/ supportive-care studies	Biomarker/ Imaging/ Molecular studies
1.	Michael Weller et al, Diagnosis and management of complications from the treatment of primary central nervous system tumors in adults, Neuro Oncol. 2023 Jul; 25(7): 1200–1224. Published online 2023 Feb 27. doi: 10.1093/neuonc/noad038		X	
2.	Anujan Poologaindran et al, Interventional neurorehabilitation for promoting functional recovery post-craniotomy: a proof-of-concept, Sci Rep. 2022; 12: 3039. Published online 2022 Feb 23. doi: 10.1038/s41598-022-06766-8	X		
3.	Kunming Cheng et al,, Emerging trends and research foci of oncolytic virotherapy for central nervous system tumors: A bibliometric study, Front Immunol. 2022; 13: 975695. Published online 2022 Sep 6. doi: 10.3389/fimmu.2022.975695			X
4.	Tawseef Ayoub Shaikh, Tabasum Rasool Dar, Shabir Sofi, A data-centric artificial intelligent and extended reality technology in smart healthcare systems, Soc Netw Anal Min. 2022; 12(1): 122. Published online 2022 Sep 1. doi: 10.1007/s13278-022-00888-7		X	
5.	Giulia Berzero et al, The coming of age of liquid biopsy in neuro-oncology. Brain. 2023 Oct; 146(10): 4015–4024. Published online 2023 Jun 8. doi: 10.1093/brain/awad195			X
6.	Sam Ng, Hugues Duffau, Brain Plasticity Profiling as a Key Support to Therapeutic Decision-Making in Low-Grade Glioma Oncological Strategies, Cancers (Basel) 2023 Jul; 15(14): 3698. Published online 2023 Jul 20. doi: 10.3390/cancers15143698	X		
7.	Matthew A et al, A systematic review of cognitive interventions for adult patients with brain tumours, Cancer Med. 2023 May; 12(10): 11191–11210. Published online 2023 Mar 7. doi: 10.1002/cam4.5760	X		
8.	Ryan P. Hamer, Tseng Tsai Yeo, Current Status of Neuromodulation-Induced Cortical Prehabilitation and Considerations for Treatment Pathways in Lower-Grade Glioma Surgery, Life (Basel) 2022 Apr; 12(4): 466. Published online 2022 Mar 22. doi: 10.3390/life12040466		X	

9.	Sé Maria Frances et al, Long-term health-related quality of life in meningioma survivors: A mixed-methods systematic review, Neurooncol Adv. 2024 Jan-Dec; 6(1): vdae007. Published online 2024 Jan 19. doi: 10.1093/noajnl/vdae007		X	
10.	Zhengang Wang et al, DNA methylation-regulated LINC02587 inhibits ferroptosis and promotes the progression of glioma cells through the CoQ-FSP1 pathway, BMC Cancer. 2023; 23: 989. Published online 2023 Oct 17. doi: 10.1186/s12885-023-11502-0			X
11.	Hugues Duffau, Repeated Awake Surgical Resection(s) for Recurrent Diffuse Low-Grade Gliomas: Why, When, and How to Reoperate?, Front Oncol. 2022; 12: 947933. Published online 2022 Jul 5. doi: 10.3389/fonc.2022.947933		X	
12.	Kazufumi Ohmura et al, Resection of positive tissue on methionine-PET is associated with improved survival in glioblastomas, Brain Behav. 2023 Dec; 13(12): e3291. Published online 2023 Oct 16. doi: 10.1002/brb3.3291			X
13.	Lydia Karamani et al, Tumor size, treatment patterns, and survival in neuro-oncology patients before and during the COVID-19 pandemic, Neurosurg Rev. 2023; 46(1): 226. Published online 2023 Sep 6. doi: 10.1007/s10143-023-02132-y	X		
14.	Sirong Song et al, Global research trends and hotspots on glioma stem cells, Front Oncol. 2022; 12: 926025. Published online 2022 Sep 29. doi: 10.3389/fonc.2022.926025			X
15.	Gambarin M, Malgrati T, Censo R Di et al. An Overview of Reviews on Predictors of Neurorehabilitation in Surgical or Non-Surgical Patients with Brain Tumours. Life 2024, Vol 14, Page 1377 2024;14(11):1377. https://doi.org/10.3390/LIFE14111377		X	
16.	Natsume K, Yoshida A, Sakakima H et al. Age-independent benefits of postoperative rehabilitation during chemoradiotherapy on functional outcomes and survival in patients with glioblastoma. Journal of Neuro-Oncology 2024 170:1 2024;170(1):129–37. https://doi.org/10.1007/S11060-024-04785-1.	X		
17.	Zanotto A, Glover RN, Zanotto T et al. Rehabilitation in People Living with Glioblastoma: A Narrative Review of the Literature. Cancers 2024, Vol 16, Page 1699 2024;16(9):1699. https://doi.org/10.3390/CANCERS16091699.	X		

18.	Natsume K, Sakakima H, Kawamura K et al. Factors Influencing the Improvement of Activities of Daily Living during Inpatient Rehabilitation in Newly Diagnosed Patients with Glioblastoma Multiforme. Journal of Clinical Medicine 2022, Vol 11, Page 417 2022;11(2):417. https://doi.org/10.3390/JCM11020417	X		
19.	Ostrom QT et al., 2022 Ostrom QT, Price M, Neff C, Cioffi G, Waite KA, Kruchko C, Barnholtz-Sloan JS. CBTRUS Statistical Report: Primary Brain and Other Central Nervous System Tumors Diagnosed in the United States in 2015-2019. Neuro Oncol. 2022 Oct 5;24(Suppl 5):v1-v95. doi: 10.1093/neuonc/noac202. PMID: 36196752; PMCID: PMC9533228.			X
20.	Das A, Ercan AB, Tabori U. An update on central nervous system tumors in germline replication-repair deficiency syndromes. Neurooncol Adv 2024;6(1). https://doi.org/10.1093/NOAJNL/VDAE102.			X
21.	Bakare, Ajibola B., Aslam, Rizwan. A systematic review of dysphagia prevalence and the role of Otolaryngologists in high-grade glioma management. Journal of Laryngology and Voice 15(1):p 10-18, January-June 2025. | DOI: 10.4103/jlv.JLV_3_25	X		
22.	Ohmura K, Daimon T, Ikegame Y *et al.* Resection of positive tissue on methionine-PET is associated with improved survival in glioblastomas. *Brain Behav* 2023;**13**(12):e3291. https://doi.org/10.1002/brb3.3291.		X	
